# Effect of dietary cadmium and/or lead on histopathological changes in the kidneys and liver of bank voles *Myodes glareolus* kept in different group densities

**DOI:** 10.1007/s10646-012-0979-z

**Published:** 2012-08-02

**Authors:** Aneta Salińska, Tadeusz Włostowski, Elżbieta Zambrzycka

**Affiliations:** 1 Institute of Biology, University of Białystok, Świerkowa 20B, 15-950 Białystok, Poland; 2 Institute of Chemistry, University of Białystok, Hurtowa 1, 15-950 Białystok, Poland

**Keywords:** Cadmium, Lead, Metallothionein, Histopathology, Lipid peroxidation, Animal density

## Abstract

Bank voles free living in a contaminated environment are known to be more sensitive to cadmium (Cd) toxicity than the rodents exposed to Cd under laboratory conditions, but the reasons for this difference are poorly defined. The present work was designed to determine whether dietary lead (Pb), a common environmental co-contaminant, and/or animal density that affects various physiological processes, would influence susceptibility to Cd toxicity in the kidneys and liver of these animals. For 6 weeks, the female bank voles were kept individually or in a group of six and provided with diet containing environmentally relevant concentrations of Cd [<0.1 μg/g (control) and 60 μg/g dry wt] and Pb [<0.2 μg/g (control) and 300 μg/g dry wt] alone or in combination. At the end of exposure period, histopathology and analyses of metallothionein, glutathione and zinc that are linked to a protective effect against Cd toxicity, as well as Cd, Pb, copper, iron and lipid peroxidation were carried out. Histopathological changes in the kidneys (a focal glomerular swelling and proximal tubule degeneration) and liver (a focal hepatocyte swelling, vacuolation and inflammation) occurred exclusively in some bank voles kept in a group and exposed to Cd alone (2/6) or Cd + Pb (4/6). The observed toxicity in grouped bank voles appeared not to be based on altered (1) tissue disposition of Cd and/or Pb, (2) metallothionein, glutathione and zinc concentrations, or (3) tissue copper, iron and lipid peroxidation. The data indicate that high population density in combination with environmental Pb may be responsible for an increased susceptibility to Cd toxicity observed in bank voles free living in a contaminated environment; the mechanism by which animal density affects Cd toxicity deserves further study.

## Introduction

Cadmium (Cd) and lead (Pb) are important toxic metals occurring in the environment naturally and as pollutants emanating mainly from industrial sources (Liu 2003; Satarug et al. 2003; Thévenod 2009). Environmental Cd has been proven to induce damage primarily to the kidneys, including tubular degeneration, interstitial inflammation, apoptosis and glomerular swelling in wildlife such as seabirds—*Fulmaris glacialis* (Nicholson et al. 1983), ptarmigan *Lagopus leucurus* (Larison et al. 2000), roe deer *Capreolus capreolus* (Beiglböck et al. 2002) and magpies *Pica pica* (Włostowski et al. 2010). Similar histopathological changes typical for Cd toxicity have been found in the kidneys of small mammals such as yellow-necked mice *Apodemus flavicolis*, bank voles, wood mice *Apodemus sylvaticus* and white-toothed shrews *Crocidura russula* free living in an area polluted with heavy metals (Damek-Poprawa and Sawicka-Kapusta 2003, 2004; Sanchez-Chardi et al. 2009). Those studies also revealed that wild animals inhabiting polluted sites are more susceptible to Cd toxicity than those exposed to the metal under laboratory conditions. For example, in the free-living bank voles from an industrialized area renal injury occurs at the Cd level lower than 20 μg/g wet wt (Damek-Poprawa and Sawicka-Kapusta 2004), while in the laboratory voles the nephropathy is evident only when the Cd concentration exceeds 50 μg/g wet wt (Włostowski et al. 2004). Still, the reason for this difference in sensitivity to Cd intoxication remains to be determined.

Noteworthy, in small mammals inhabiting an industrialized area not only accumulation of Cd but also appreciable concentrations of Pb in the kidneys have been detected (Damek-Poprawa and Sawicka-Kapusta 2003, 2004). It is also known from animal studies that chronic exposure to Pb can lead to nephropathy, including nephromegaly and dysfunction of proximal tubules (Liu et al. 2012; Qu et al. 2002). In addition, combined exposure to Pb and Cd has been shown to exacerbate cytotoxicity in the rat proximal tubular cells (Wang et al. 2011). However, it is unknown whether environmental Pb can produce or only enhance existing Cd-induced renal damage in the wild animals (Damek-Poprawa and Sawicka-Kapusta 2003, 2004).

It is important to point out that free-ranging animals, compared to those raised in laboratory, are concurrently subjected to other stressful conditions, including changes in food resources, threats from predators, and interaction with conspecifics (Beiglböck et al. 2002; Damek-Poprawa and Sawicka-Kapusta 2004; Pollock and Machin 2009; Tidhar et al. 2007). In general, every individual encounters stress originating from interaction with conspecifics, especially when population density is high (Marchlewska-Koj et al. 1994; Pollock and Machin 2009). It has been demonstrated that the psychosocial stress resulting from crowding can modify growth, reproduction, behavior, immune function and other physiological processes in various species, including the bank vole (Grippo et al. 2010; Marchlewska-Koj et al. 1994, 2003; Tort et al. 1996; Vicario et al. 2012). However, the role of animal density or interaction with conspecifics in Cd toxicity remains unknown.

Therefore, the present work was designed to determine whether dietary Pb and/or animal density would influence Cd toxicity in the kidneys and liver of bank voles [*Myodes (*=*Clethrionomys) glareolus*] that appeared to be vulnerable to the metal toxicity when free living in a contaminated area (Damek-Poprawa and Sawicka-Kapusta 2004). The toxicity was evaluated by assessing kidney and liver histopathology. In addition, analyses of metallothionein (MT), glutathione (GSH), Cd, Pb, zinc (Zn), copper (Cu), iron (Fe) and lipid peroxidation were carried out to find out whether Pb and/or animal density affect sensitivity to Cd toxicity (if any) by modulating MT induction, GSH metabolism, Cd kinetics or trace element concentrations and oxidative stress. All the examined processes and elements are commonly thought to be important ones during development of Cd toxicity. Specifically, MT, GSH and Zn are linked to a protective effect against Cd toxicity (Chan and Cherian 1992; Jacquillet et al. 2006; Jihen et al. 2008; Klaassen et al. 2009; Tang et al. 1998), while Cu, Fe and oxidative stress are considered to be responsible for the progression of toxicity (Liu et al. 2009; Thévenod and Friedmann 1999; Whittaker et al. 2011).

## Materials and methods

### Animals and experimental design

The bank vole is a common European rodent that has become a model for ecological and ecotoxicological experiments. Field and laboratory studies revealed that population density affects particularly female voles, including their reproduction and behavior (Marchlewska-Koj et al. 1994, 2003). Therefore, female bank voles (1 month old, weighing 11–13 g), being the F_1_ offspring of the wild-caught stock (Knyszyn Old Forest, north-eastern Poland) were used throughout the study. The bank voles were randomly divided into two groups according to the cage density: (1) one vole per cage [low density group (LD)], and (2) six voles per cage [high density group (HD)]. At the beginning of the experiment each density group was divided into four subgroups (*n* = 6 each) according to dietary Cd and Pb: (1) control, (2) Cd—60 μg/g, (3) Pb—300 μg/g, and (4) Cd—60 μg/g + Pb—300 μg/g dry wt. The animals were housed for 6 weeks in stainless-steel cages (44 × 27 × 20 cm) (lined with peat as absorptive material) at 18–20 °C on 12 light/dark cycle and at 50–70 % relative humidity. They received ad libitum distilled water and control or Cd- and Pb-containing whole wheat grains, which appeared to be an adequate quality food for these rodents (Włostowski et al. 2004). In addition, an identical quantity of apple was offered to all voles (3 g/vole/week), who ate it completely. The food intake was monitored throughout the experiment. Prior to the experiment the grains were contaminated with Cd and/or Pb [soaked in CdCl_2_ or Pb(NO_3_)_2_ solutions]. Atomic absorption spectrophotometry (AAS) analysis of the grains revealed that actual levels of Cd were between 58 and 63 μg/g (Cd groups) and those for Pb were 280–310 μg/g dry wt (Pb groups). Control grains contained less than 0.1 μg Cd/g and 0.2 μg Pb/g. The concentrations of Zn, Cu and Fe in the grains were 22–26, 4–6 and 80–100 μg/g dry wt, respectively. The concentrations of dietary Cd and Pb were two-fold higher than those observed in a heavily contaminated environment (Liu 2003) and chosen to obtain more pronounced results.

At the end of the 6-week exposure period, the bank voles were weighed, killed by decapitation and the liver and kidneys were removed, rinsed in cold saline, and blotted dry on absorbent paper. Blood was also taken to determine hemoglobin and hematocrit by using standard methods (spectrophotometrically as cyanmethemoglobin at 540 nm and hematocrit centrifuge, respectively). One kidney and a portion of the fresh liver (0.25 g) were transferred to 1.0 ml chilled 0.25 M sucrose and homogenized with a Teflon pestle in a glass homogenizer. Aliquots (0.5 and 0.1 ml) of the homogenate were taken for determination of metal concentrations and lipid peroxidation, respectively. The remaining homogenate was centrifuged at 20,000×*g* for 20 min at 4 °C, and the resulting supernatant was removed for MT and GSH assays.

### Histological examination

One kidney and a portion of the liver were fixed in 4 % formaldehyde, dehydrated in ethanol and xylene, embedded in paraffin, cut into 5-μm sections, and stained with hematoxylin and eosin for microscopic examination.

### Metal determination

Metal determinations were performed as described recently (Salińska et al. 2012). The homogenate (0.5 ml) was placed in a glass tube with 2.0 ml of concentrated nitric acid. After 20 h of sample digestion at room temperature, 72 % perchloric acid (0.5 ml) was added and the mixture was heated at 100 °C for 3 h. Finally, the temperature was raised to 150–180 °C and digestion continued for another 2 h. Deionized water was added to the residue (0.1 ml) after digestion to a volume of 3.0 ml (first solution). A portion of the first solution (200 μl) was evaporated to dryness in a quartz crucible at 130 °C, and the residue was redissolved in an appropriate amount of deionized water (second solution). Cd and Pb analyses of these solutions were carried out by electrothermal AAS using a Solaar M6 instrument with a Zeeman correction. The concentrations of Zn, Cu and Fe in the first solution were determined by AAS in an air-acetylene flame with a deuterium correction. Quality assurance procedures included the analysis of reagent blanks and appropriate standard reference material (NIST bovine liver 1577b). The recovery of Cd, Pb, Zn, Cu and Fe were 91–93, 105–115, 90–95, 89–95 and 95–101 %, respectively.

### MT determination

MT in the kidneys and liver was determined by a Cd-saturation method (Włostowski et al. 2004). Briefly, a 0.1 ml sample was incubated in a 1.5-ml vial for 10 min at room temperature with 1.0 ml Tris–HCl buffer (0.03 M, pH 7.8) containing 1.0 μg Cd/ml. To remove non-MT-bound Cd, bovine hemoglobin (Sigma) (0.1 ml of a 5 % solution in H_2_O) was added and the sample was heated for 1.5 min at 95 °C, cooled, and centrifuged for 5 min at 10.000×*g*. Addition of hemoglobin, heating, and centrifugation of the sample was repeated twice. Cd bound to MT in the resulting clear supernatant was determined by electrothermal AAS. MT content was expressed in μg of the protein per gram of wet tissue, assuming that 1 mol of MT (6600) binds 7 mol of Cd. Cd bound to MT in the kidneys and liver was determined by using the same method but without Cd saturation. Cd not bound to MT was defined as the difference between the total tissue Cd and the Cd bound to MT.

### GSH assay

The total GSH (reduced + oxidized) was measured in the postmitochondrial fraction according to the method of Tietze (1969) by using NWLSS Glutathione Assay Kit (Vancouver, WA, USA). Briefly, an aliquot of the supernatant (50 μl) was deproteinized by adding 100 μl of an aqueous solution of 5 % metaphosphoric acid. After centrifugation an aliquot (25 μl) of the supernatant was diluted by adding 500 μl of assay buffer. To 400 μl of this solution 400 μl of assay buffer, 50 μl of 5,5′-dithiobis-2-nitrobenzoic acid (DTNB) and 50 μl of GSH reductase in assay buffer were added and incubated for 2 min and 30 s at room temperature. Subsequently 50 μl of NADPH solution was added and the reduction rate of DTNB into 5-thio-2-nitrobenzoic acid (TNB) was measured spectrophotometrically at 412 nm for 3 min. GSH was expressed as μmol/g wet weight.

### Lipid peroxidation assay

Lipid peroxidation was assessed by measuring malondialdehyde formation, using the thiobarbituric acid assay (Ohkawa et al. 1979). To 0.1 ml of the tissue homogenate, 0.2 ml of 8.1 % sodium dodecyl sulfate, 1.5 ml of 20 % acetic acid, 1.5 ml of 0.8 % TBA and 0.6 ml of distilled water were added and vortexed. The reaction mixture was placed in a water bath at 95 °C for 1 h. After cooling, 1.0 ml of distilled water and 5.0 ml of butanol/pyridine mixture (15:1 v/v) were added and vortexed. After centrifugation, absorbance of the organic phase was determined at 532 nm. Tetraethoxypropane was used to prepare a calibration curve. The results were expressed as TBA-reacting substances (nmol/g wet weight).

### Statistical analysis

Data were expressed as mean ± SD. The effects of Cd and Pb were tested by means of two-way analysis of variance (ANOVA) in the bank voles kept individually (Group 1) or in a group of six (Group 2). Differences between the groups and subgroups were analyzed by one-way ANOVA followed by the Duncan’s multiple range test (SPSS 14.0). Differences at *P* < 0.05 were considered statistically significant.

## Results

Subchronic consumption of dietary Cd and Pb alone or in combination had no effect on body weight (Table 1) and food intake (0.15–0.18 g/g body wt/day) in the bank voles kept individually (LD voles) or in a group of six (HD voles). Although enlarged kidneys are indicative of chronic Cd and Pb toxicity (Liu et al. 2000; Qu et al. 2002), in the present study subchronic Cd and Pb alone or in combination did not affect the kidneys or liver weights in bank voles from the two cage density groups (Table 1). Despite the fact that exposure to Cd and Pb can result in decreased hemoglobin and hematocrit values (Whittaker et al. 2011; Włostowski et al. 2000), no changes of these indices were observed in the LD- and HD bank voles exposed to dietary Cd and/or Pb (Table 1).

**Table 1:** Body and organ weights, hematological values, and incidence of histopathological changes in the kidneys and liver of female bank voles exposed to dietary Cd and/or Pb and raised individually or in a group of six

Subgroup	Bady mass (g)	Kidneys mass (g)	Liver mass (g)	Hemoglobin (g/100 ml)	Hematocrit (%)
Group 1: One vole per cage (six cages/subgroup)					
Control	14.9 ± 0.7	0.16 ± 0.01 (0/6)	0.55 ± 0.04 (0/6)	15.3 ± 1.1	48.0 ± 2.4
Cd	15.1 ± 1.7	0.17 ± 0.02 (0/6)	0.69 ± 0.18 (0/6)	16.8 ± 1.0	50.1 ± 2.1
Pb	15.3 ± 1.8	0.18 ± 0.01 (0/6)	0.62 ± 0.11 (0/6)	16.7 ± 1.3	50.9 ± 1.4
Cd + Pb	16.4 ± 0.9	0.18 ± 0.01 (0/6)	0.66 ± 0.09 (0/6)	16.6 ± 1.4	51.7 ± 2.9
Group 2: Six voles per cage (one cage/subgroup)					
Control	15.6 ± 0.8	0.16 ± 0.01 (0/6)	0.57 ± 0.04 (0/6)	15.5 ± 1.0	49.0 ± 2.0
Cd	15.1 ± 0.5	0.17 ± 0.01 (2/6)	0.51 ± 0.03 (2/6)	15.1 ± 0.7	48.3 ± 1.5
Pb	15.2 ± 0.7	0.17 ± 0.01 (0/6)	0.60 ± 0.08 (0/6)	16.2 ± 1.1	49.0 ± 2.5
Cd + Pb	15.9 ± 0.8	0.18 ± 0.02 (4/6)	0.59 ± 0.11 (4/6)	15.9 ± 1.3	50.1 ± 2.0

**Fig. 1:**
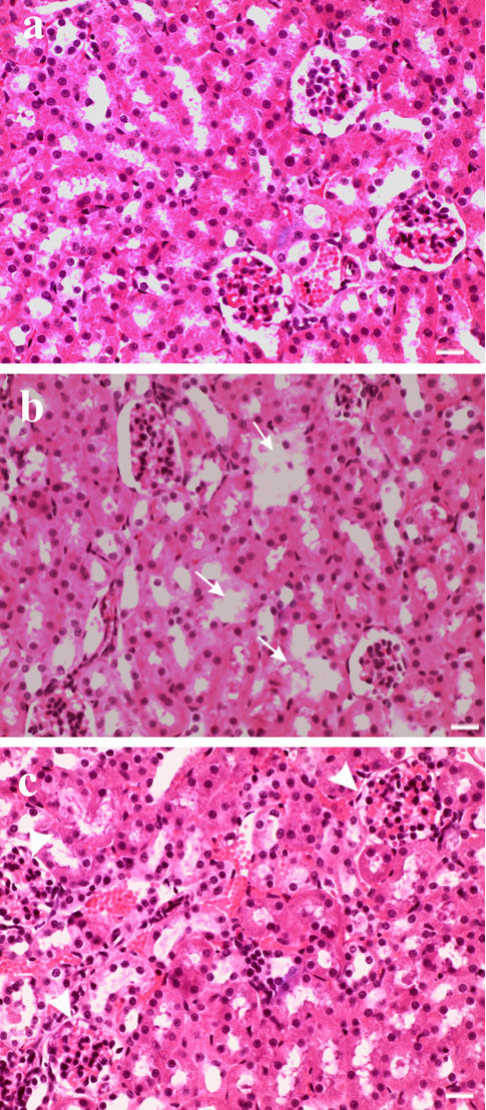
Representative photomicrographs of kidney section from (**a**) control bank voles, and (**b**, **c**) bank voles raised in a group of six and exposed to dietary Cd [**b** tubule degeneration (*arrows*), **c** glomerular swelling (*arrowhead*)]. *Scale bar*, 20 μm

**Fig. 2:**
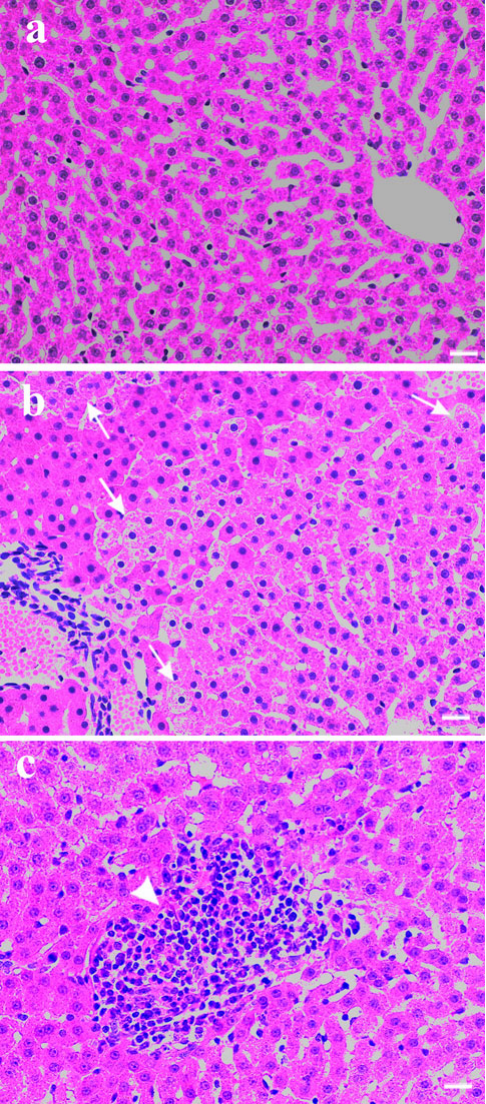
Representative photomicrographs of liver section from (**a**) control bank voles, and (**b, c**) bank voles raised in a group of six and exposed to dietary Cd [**b** hepatocyte swelling (*arrows*), **c** leukocyte infiltration (*arrowhead*)]. *Scale bar*, 20 μm

Values represent the mean ± SD for *n* = 6. Bank voles received, for 6 weeks, control diet or diets containing 60 μg Cd/g and/or 300 μg Pb/g. In parentheses the number of voles with histopathological changes per total number of animals is presented (see Figs. 1, 2). There were no statistically significant differences between the groups and subgroups

The histopathological changes in the kidneys and liver are shown in Figs. 1 and 2, respectively. The kidneys and liver of control LD- and HD bank voles showed normal morphology (Figs. 1a, 2a). Likewise, the normal morphology was found in all LD bank voles exposed to Cd and/or Pb (Table 1). In contrast, treatment with Cd alone produced a focal proximal tubule degeneration (Fig. 1b) and glomerular swelling (Fig. 1c) in the kidneys, and a focal hepatocyte swelling (Fig. 2b), vacuolation and inflammation (leukocyte infiltration) (Fig. 2c) in the liver of two (per six) HD voles (Table 1). The same changes were observed in four (per six) HD animals exposed simultaneously to Cd and Pb; a normal histological picture of the kidneys and liver was noted in the grouped bank voles exposed only to dietary Pb (Table 1). Despite the occurrence of histopathology, renal and hepatic lipid peroxidation was not significantly affected by Cd, Pb and interaction between these metals (*P* > 0.1) in the two density groups (Tables 2, 3).

**Table 2:** Metallothionein and metal concentrations, and lipid peroxidation (TBARS) in the kidneys of female bank voles exposed to dietary Cd and/or Pb and raised individually or in a group of six

Subgroup	Metallothionein (μg/g wet wt)	Cadmium (μg/g wet wt)	Zinc (μg/g wet wt)	Copper (μg/g wet wt)	Iron (μg/g wet wt)	Lead (μg/g wet wt)	TBARS (nmol/g wet wt)
Group 1: One vole per cage (six cages/subgroup)							
Control	14.9 ± 3.0a	0.36 ± 0.08a	15.7 ± 4.2a	5.2 ± 0.7a	102 ± 10a	0.56 ± 0.10a	142 ± 30a
Cd	311 ± 25b	33.2 ± 6.7b (9.0)	29.5 ± 3.8b	5.0 ± 0.4a	95 ± 11a	0.53 ± 0.18a	144 ± 17a
Pb	15.6 ± 1.5a	0.37 ± 0.07a	16.0 ± 2.6a	5.4 ± 0.9a	96 ± 18a	12.7 ± 1.2b	135 ± 30a
Cd + Pb	300 ± 15b	28.1 ± 7.2b (7.0)	26.2 ± 3.0b	5.2 ± 0.3a	92 ± 15a	11.2 ± 2.0b	160 ± 15a
Group 2: Six voles per cage (one cage/subgroup)							
Control	14.2 ± 3.2a	0.35 ± 0.05a	15.4 ± 3.7a	5.1 ± 0.7a	92 ± 15a	0.49 ± 0.15a	140 ± 10a
Cd	293 ± 31b	32.0 ± 4.2b (8.9)	26.3 ± 3.6b	5.7 ± 0.8a	91 ± 10a	0.56 ± 0.11a	130 ± 12a
Pb	15.3 ± 2.5a	0.35 ± 0.06a	16.5 ± 2.5a	5.4 ± 0.5a	94 ± 12a	13.1 ± 2.5b	139 ± 15a
Cd + Pb	297 ± 30b	34.2 ± 6.7b (8.5)	25.0 ± 3.5b	5.3 ± 0.4a	88 ± 10a	10.5 ± 2.0b	150 ± 21a

Values represent the mean ± SD for *n* = 6. Bank voles received, for 6 weeks, control diet or diets containing 60 μg Cd/g and/or 300 μg Pb/g. In parentheses mean concentration of Cd not bound to MT is shown. Means in the same column marked with a different letter are significantly different (*P* < 0.05)

**Table 3:** Metallothionein, glutathione and metal concentrations, and lipid peroxidation (TBARS) in the liver of female bank voles exposed to dietary Cd and/or Pb and raised individually or in a group of six

Subgroup	Metallothionein (μg/g wet wt)	Cadmium (μg/g wet wt)	Zinc (μg/g wet wt)	Copper (μg/g wet wt)	Iron (μg/g wet wt)	Lead (μg/g wet wt)	TBARS (nmol/g wet wt)	Glutathione (μmol/g wet wt)
Group 1: One vole per cage (six cages/subgroup)								
Control	4.9 ± 0.4a	0.13 ± 0.02a	23.2 ± 2.1a	3.6 ± 0.2a	316 ± 128a	0.35 ± 0.15a	108 ± 32a	6.5 ± 2.0a
Cd	238 ± 66b	21.8 ± 5.4b (5.7)	34.5 ± 5.1b	3.7 ± 0.4a	110 ± 30b	0.33 ± 0.10a	93 ± 8a	7.5 ± 1.6a
Pb	4.8 ± 0.5a	0.15 ± 0.02a	24.7 ± 2.6a	3.8 ± 0.7a	349 ± 130a	1.90 ± 0.50b	101 ± 17a	6.2 ± 2.2a
Cd + Pb	235 ± 15b	20.5 ± 3.0b (5.9)	34.2 ± 5.6b	3.5 ± 0.5a	135 ± 45b	1.80 ± 0.50b	95 ± 10a	7.5 ± 1.5a
Group 2: Six voles per cage (one cage/subgroup)								
Control	5.1 ± 0.5a	0.12 ± 0.02a	24.2 ± 4.5a	3.8 ± 0.5a	237 ± 60a	0.30 ± 0.10a	106 ± 17a	6.7 ± 3.0a
Cd	243 ± 19b	20.7 ± 3.9b (5.2)	34.2 ± 4.9b	3.9 ± 0.6a	100 ± 25b	0.35 ± 0.11a	96 ± 11a	7.2 ± 2.0a
Pb	5.0 ± 0.6a	0.14 ± 0.03a	23.9 ± 2.1a	3.8 ± 0.4a	300 ± 70a	1.85 ± 0.70b	108 ± 10a	6.4 ± 1.8a
Cd + Pb	260 ± 34b	21.0 ± 4.1b (5.9)	36.3 ± 5.5b	3.7 ± 0.3a	130 ± 35b	1.70 ± 0.65b	97 ± 15a	7.4 ± 2.2a

Values represent the mean ± SD for *n* = 6. Bank voles received, for 6 weeks, control diet or diets containing 60 μg Cd/g and/or 300 μg Pb/g. In parentheses mean concentration of Cd not bound to MT is shown. Means in the same column marked with a different letter are significantly different (*P* < 0.05)

The concentrations of renal and hepatic MT were significantly affected only by dietary Cd (*P* < 0.0001) (Tables 2, 3). The MT levels in the kidneys and liver of HD bank voles did not differ from those of the respective LD animals. Likewise, the accumulation of Cd in the kidneys and liver was also influenced only by dietary Cd (*P* < 0.0001) and reached similar level in the LD- and HD bank voles (Tables 2, 3). Assuming that 1 mol of MT (6600) binds 7 mol of Cd, the Cd-binding capacity of MT exceeded the total concentration of Cd by 1–8 μg/g in the kidneys and by 7–10 μg/g in the liver. Despite this, the fraction of renal and hepatic Cd not bound to MT amounted to 25–30 % of the total tissue Cd in all bank voles exposed to dietary Cd or Cd + Pb (Tables 2, 3).

Neither dietary Cd alone and Pb alone nor in combination affected significantly (*P* > 0.1) the concentration of GSH in the liver of bank voles from the two density groups (Table 3). The renal GSH could not be detected by the method used, probably because of too small sample mass.

Subchronic consumption of Cd, either alone or in combination with Pb, increased significantly (*P* < 0.0000) Zn concentrations in the kidneys and liver of bank voles from the two density groups (Tables 2, 3). In general, the tissue Zn followed a pattern similar to that of MT concentration. The renal and hepatic Cu as well as the renal Fe were not affected (*P* > 0.1) by dietary Cd and/or Pb, whereas the hepatic Fe decreased 2–3-fold upon the exposure to Cd alone or Cd + Pb in the LD- and HD bank voles (Tables 2, 3). In addition, the concentrations of Pb in the kidneys and liver of bank voles exposed to Pb alone or in combination with Cd were similar (*P* > 0.1) in the two density groups, but the renal Pb was six-fold higher than that in the liver (Tables 2, 3).

## Discussion

The present study demonstrates that relatively high dietary Cd alone produces histopathological changes in the kidneys and liver of some bank voles kept in a group but not in those raised individually. In contrast, dietary Pb alone does not induce any renal and hepatic injury in the two density groups but in combination with Cd appears to increase the frequency of Cd-induced histopathological changes only in the grouped animals. These findings suggest that high population density or interaction with conspecifics in combination with environmental Pb may increase susceptibility to Cd toxicity observed in bank voles free living in a contaminated environment (Damek-Poprawa and Sawicka-Kapusta 2004).

There are several potential explanations for the nephro- and hepatotoxicity developed during exposure to Cd alone or co-exposure to Cd and Pb exclusively in the grouped bank voles, including toxicokinetic and toxicodynamic interactions. However, the toxicity does not appear to occur at the toxicokinetic level, as tissue accumulation of both Cd and Pb was similar, when given alone or together, in bank voles raised in a group or individually (Tables 2, 3). Likewise, the renal and hepatic MT, a primary component of acquired tolerance to toxic effects of Cd (Klaassen et al. 2009), as well as the fraction of non-MT-bound Cd [thought as the toxic species (Goyer et al. 1989; Sudo et al. 1996)] were similar in the respective voles from the two density groups. These findings exclude the possibility that the occurrence of histopathology in the grouped animals was due to insufficient amount of the protein or too high content of the non-MT-bound Cd. Similarly, GSH that is known to provide protection against Cd toxicity (Chan and Cherian 1992) could have only a negligible effect because its content was relatively stable in all bank voles exposed to Cd and/or Pb (Table 3). It seems also unlikely that the increased kidney and liver injury from Cd and Pb co-exposure in the grouped bank voles was owing to increased oxidative stress [thought to be a cellular mechanism of toxicity (Thévenod and Friedmann 1999; Wang et al. 2011)], as the renal and hepatic lipid peroxidation as well as the concentrations of prooxidant elements such as Cu and Fe (Whittaker et al. 2011) were similar in respective animals from the two groups. Furthermore, renal and hepatic Zn that is well known as an antioxidant and protects against Cd toxicity (Jacquillet et al. 2006; Jihen et al. 2008), increased to the same level in the grouped and individual bank voles exposed to dietary Cd alone or in combination with Pb (Tables 2, 3). This implies that the fraction of Zn, which most likely was sequestered by MT [specifically by binding sites not occupied by Cd (see “Results” section)], was ineffective in protection against the toxicity. Overall, although the precise basis for the nephro- and hepatotoxicity produced by Cd alone or in combination with Pb only in the grouped bank voles cannot be defined from the present work, it does not appear to be based on altered (1) tissue disposition of Cd and/or Pb, (2) MT, GSH, and Zn concentrations, or (3) tissue Cu, Fe and lipid peroxidation.

The present study also indicates that environmentally relevant concentration of dietary Pb only increases the frequency of Cd-induced damage to the kidneys and liver of grouped bank voles. This remains in some contrast to other studies indicating that Cd and Pb can produce toxicity in additive or synergistic manner both in vivo and in vitro (Wang et al. 2010, 2011). It is also evident from those studies that oxidative stress may be induced by the two metals. In this regard, Cd and Pb co-exposure produces significantly more lipid peroxidation in the kidneys of rats than either inorganic given alone (Wang et al. 2010). In the present work Cd alone and Pb alone or in combination did not affect lipid peroxidation in the kidneys and liver of bank voles, suggesting no involvement of this process in the injury. Other studies have also contested the main role of lipid peroxidation in cell injury induced by Cd (Stacey et al. 1980; Włostowski et al. 2000, 2010). Sill, these data do not exclude the possibility that the two metals could induce oxidative stress, but the process could not be revealed by using only TBARS assay, which appears to have some limitations, especially when the whole tissue is examined (Meagher and FitzGerald 2000).

Although the cause for the observed difference in susceptibility to Cd-induced renal and hepatic injury between the two density groups of bank voles remains unknown, it cannot be ruled out that the difference could potentially be associated with the negative interaction between animals within a group. It is well known that the interaction with conspecifics can induce psychosocial stress, resulting in various pathophysiological processes (Grippo et al. 2010; Marchlewska-Koj et al. 1994; Tort et al. 1996; Vicario et al. 2012). Specifically, the stress has been shown to alter morphology of mitochondria (swelling and loss of cristae) as well as the activity of citrate synthase, leading to less number of viable mitochondria (Vicario et al. 2010, 2012). Interestingly, similar changes in the morphology of mitochondria were observed in the kidneys of rats upon exposure to Cd and/or Pb (Wang et al. 2010). Thus, it is reasonable to assume that the combined exposure to the stress and metals could exacerbate the injury to mitochondria, resulting in pronounced reduction of ATP synthesis. An ATP deprivation, in turn, could cause the loss of ionic control and the cellular disintegration (Cannino et al. 2009; Nicholson et al. 1983); the presence of hepatocyte swelling (Fig. 2b) in the grouped bank voles exposed to Cd alone or Cd + Pb might support the assumption. However, further investigations are needed to clarify the role of psychosocial stress in the metal toxicity in bank voles and other species.

In summary, the results of the present work indicate that high animal density in combination with dietary Pb markedly exacerbates Cd toxicity in the kidneys and liver of bank voles. This effect does not appear to involve altered toxicokinetic as well as tissue trace element and lipid peroxidation, and occurs despite sufficiently high concentrations of MT. The mechanism by which animal density affects Cd toxicity deserves further study.
